# Herlyn-werner-wunderlich syndrome: MRI findings, radiological guide (two cases and literature review), and differential diagnosis

**DOI:** 10.1186/1471-2342-12-4

**Published:** 2012-03-09

**Authors:** Riccardo Del Vescovo, Sofia Battisti, Valerio Di Paola, Claudia L Piccolo, Roberto L Cazzato, Ilaria Sansoni, Rosario F Grasso, Bruno Beomonte Zobel

**Affiliations:** 1 Department of Radiology, Campus Bio-Medico Univeristy of Rome, Italy

## Abstract

**Background:**

Herlyn-Werner-Wunderlich (HWW) syndrome is a very rare congenital anomaly of the urogenital tract involving Müllerian ducts and Wolffian structures, and it is characterized by the triad of didelphys uterus, obstructed hemivagina and ipsilateral renal agenesis. It generally occurs at puberty and exhibits non-specific and variable symptoms with acute or pelvic pain shortly following menarche, causing a delay in the diagnosis. Moreover, the diagnosis is complicated by the infrequency of this syndrome, because Müllerian duct anomalies (MDA) are infrequently encountered in a routine clinical setting.

**Cases presentation:**

two cases of HWW syndrome in adolescents and a differential diagnosis for one case of a different MDA, and the impact of magnetic resonance (MR) imaging technology to achieve the correct diagnosis.

**Conclusions:**

MR imaging is a very suitable diagnostic tool in order to perform the correct diagnosis of HWW syndrome.

## Background

The association of renal agenesis with ipsilateral blind hemivagina and didelphys uterus is reported as Herlyn-Werner-Wunderlich (HWW) syndrome; this triad was initially disclosed in an English report published in 2006 [1].

The incidence of didelphys uterus, related to HWW, is approximately 1/2,000 to 1/28,000, and it is accompanied by unilateral renal agenesis in 43% of cases. The incidence of unilateral renal agenesis is 1/1,100, and 25-50% of affected women exhibits associated genital abnormalities [2].

HWW syndrome represents a type of Müllerian duct anomalies (MDA) associated with mesonephric duct anomalies. MDA are congenital entities resulting from non-development (agenesia or hypoplasia), defective vertical or lateral fusion, or resorption failure of the Müllerian (paramesonephric) ducts [3].

MDA are estimated to have an overall prevalence of 2% to 3% among all women, with an incidence of 1/200-600 among fertile women. Hypoplasia, as well as agenesis of the uterus and proximal vagina, accounts for 5%-10% of Müllerian duct anomalies, whereas didelphys uterus accounts for approximately 11% of Müllerian duct anomalies. Renal tract anomalies are associated with MDA in as many as 30% of cases [4].

A complete or partial vaginal septum is present in 75% of women with didelphys uterus [5].

The exact cause, pathogenesis and embryologic origin of HWW syndrome are unclear and remain a subject of discussion [6].

HWW syndrome is usually discovered at puberty with non-specific symptoms, like increasing pelvic pain, dysmenorrhea and palpable mass due to the associated haematocolpos or hematometra, which result from retained, longstanding menstrual flow in the obstrucucted vagina.

It rarely occurs with primary infertility in early adulthood when the vaginal septum is incomplete [7].

It is really difficult to achieve to an accurate diagnosis because menstruation is often regular and when patient complains symptoms of cyclic dysmenorrhea, they are usually given anti-inflammatory drugs and oral-contraceptives, thus causing a delay in the diagnosis because they reduce or eliminate menses; ultimately, HWW is an uncommon syndrome, not often thought of as a diagnostic possibility [8].

The potential complications of this syndrome are distinct in acute complications, such as pyohematocolpos, pyosalpinx, or pelviperitonitis, and long-term complications, such as endometriosis, pelvic adhesions and increased risk of abortion or infertility [9,10].

The American Society for Reproductive Medicine (ASRM) established a classification of Müllerian duct anomalies in 1988 in order to group them in entirety (Table 1) [11].

**Table 1:** Classification of müllerian duct anomalies

MULLERIAN AGENESIS OR HYPOLPLASIA	a) vagina b) cervix c) fundus d) fallopian tube e) combined agenesis or hypoplasia (two or more findings from classes I-A through I-D)
**UNICORNUATE UTERUS **(Agenesis or hypoplasia of one of the two Mullerian ducts)	• rudimentary horn with an endometrial cavity that communicates with the (single-horned) uterus; • rudimentary horn with an endometrial cavity that does not communicate with the uterus; • rudimentary horn with no endometrial cavity; no rudimentary horn

**UTERUS DYDELPHYS** (Failure of lateral fusion between vagina and two Mullerian ducts)	

**BICORNUATE UTERUS** (Incomplete fusion of uterine horns at the level of the fundus)	a) complete (septum extends to the internal or external os); b) partial (septum is confined to the fundal region)

**SEPTATE UTERUS** (Incomplete or absent resorption of uterovaginal septum)	a) complete (septum extends to the internal os); b) partial (septum does not reach the internal os)

**ARCHUATE UTERUS** (Light indent of the fundus of the uterus due to almost complete resorption of the uterovaginal septum)	

**UTERUS EXPOSED TO DIETHYILSTILBESTROL-RELATED UTERINE ANOMALIES**	a) T-shaped uterus; b) T-shaped uterus with dilated horns; c) Uterine hypoplasia

According to this classification, HWW syndrome appears to include the addition of III uterine anomaly to Ia vaginal anomaly and renal agenesis (Table 2).

**Table 2:** Hww syndrome: mr findings

HWW SYNDROME	MR FINDINGS
Uterus didelphys	It is characterized by two symmetric, widely divergent uterine horns and two cervixes, with an enlarged cavity filled by bloody/proteinaceous fluid due to haematocolpos. It appears hyperintense signal on T1 fat saturated sequences.

Hemivagina	It is an obstructed vagina due to a longitudinal septum which occludes one cervix and isolates it with consequent hematometra.

Renal aplasya	It is tipically omolateral to the vaginal anomaly (right side prevalence), with possible controlateral renal hypertrophy due to compensation but in site.

Ovaries	Possible presence of endometriosic cysts with hyperintense signal on T1 fat sat sequences and with "shading sign" on T2-weighted images and a gradual variation of signal intensity due to chronic bleeding with accumulation of high concentration of iron and protein in the endometrioma (endometriotic lesions appear hyperintense on T1-weighted images and mildly hypointense or hyperintense on T2-weighted images). Possible presence of functional cysts with no clinical significance, which appear hyperintense on T2 sequences and hypo/isointense on T1 sequences.

## Case presentation

### Patient 1

A 16-years-old female presented with a history of severe abdomino-pelvic pain, which increased lasting 2 to 7 days with each of her menstrual cycles, hindering her daily activities.

Gynecologic history indicated menarche at 13 years of age, followed by 3 years of irregular menses.

The patient denied any recent abdominal trauma, abnormal vaginal bleeding, nausea, vomiting or diarrhea.

A bimanual physical examination indicated a right-sided cystic and tender pelvic mass, movable, mildly tender to palpation. The medical staff recommended further evaluation such as US, which showed the absence of right kidney and the possibility of uterine anomaly; so Magnetic Resonance Imaging (MRI) was performed in order to evaluate the possible genito-urinary anomaly.

The MRI examination was performed on a 1.5 Tesla MR clinical scanner (Siemens, Avanto Erlangen Germany). The patient's mother gave her informed consent for MRI exam. Images were acquired on multiple planes with T1-weighted (TR 714 ms; TE 12 ms) and T2-weighted Turbo Spin Echo (TR 5530 ms; TE 150 ms) sequences; fat-suppressed T2-weighted TSE images (TR 6810 ms; TE 150 ms), Flash3D T1-weighted (TR 5.2; TE 2.5) and HASTE sequences (TR 700; TE 89). A contrast agent was not used as it was not considered necessary.

**Figure 1:**
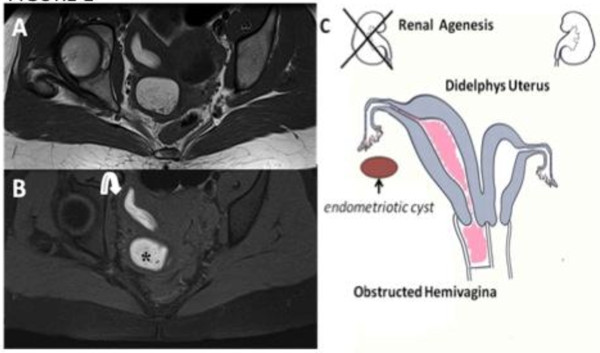
**Herlyn-Werner-Wunderlich syndrome (C) Drawing illustrates the triad (renal agenesis, didelphys uterus, and obstructed hemivagina and in this case the presence of an endometriosic cyst**. A 16-years-old girl presenting the triad of didelphys uterus (class III MDA), an obstructed right hemivagina (class I MDA), and ipsilateral renal agenesis. (A) Axial turbo spin-echo T2 weighted (B) Axial turbo spin-echo T1 fat-saturated weighted MR image showing centrally a hematocolpos (asterisk), a finding corresponding to the obstructed right hemivagina. Mild dilation of the right endometrial cavity (curved arrow) with fluid exhibiting a signal intensity similar to methaemoglobin due to haematometra.

MRI imaging showed a uterine-vaginal anomaly consisting of didelphys uterus and double vagina, one of which was obstructed (Figure 1); consequently, there was accumulation of fluid exhibiting a signal intensity similar to methaemoglobin in the right uterus (slightly dilated, 14 mm diameter of the lumen) and in the right obstructed vagina (which appeared to be considerably dilated, 43 × 34 × 35 mm).

**Figure 2:**
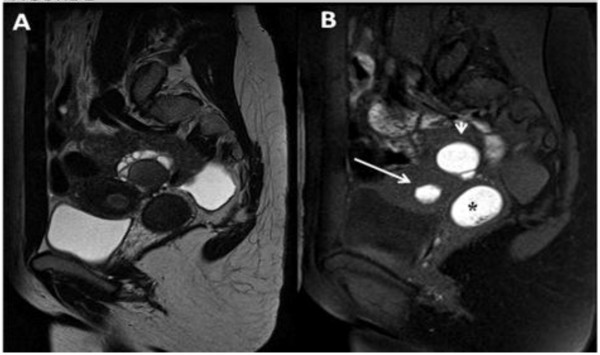
**A) Sagittal T2 TSE weighted image showing a mild fluid in the pouch of Douglas**. (B) Sagittal T1 TSE fat-suppressed weighted image showing distention of the right endometrial cavity (long straight arrow) with high-signal-intensity material distending the right endometrial cavity and cervix (asterisk), a finding indicative of right hematometrocolpos; on the right ovary presence of an endometriosic cysts appearing hyperintense on T1 fat-saturated image (short straight arrow).

In the right ovary an expanding mass of 31 mm was hyperintense on T1-weighted images and mildly hypointense or hyperintense on T2-weighted images (Figure 2A). The mass was characterized by a typical "shading" sign (Figure 2B) and exhibited features compatible with an endometriosic cyst; there was mild fluid in the pouch of Douglas.

**Figure 3:**
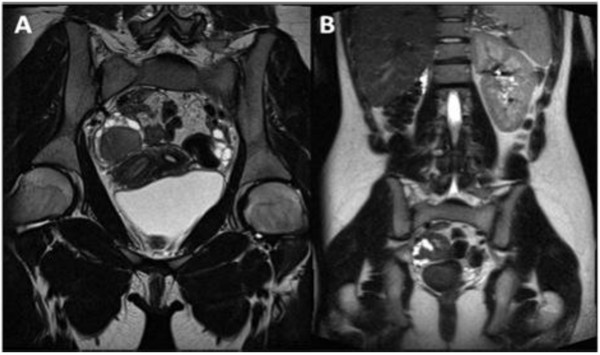
**A) Oblique coronal TSE T2-weighted MR image shows a complete view of the anatomical defects**. B) Coronal T2 Trufi MR image showing the omolateral renal aplasia with respect to the pelvic abnormalities and a solitary left kidney with a mild hypertrophy.

There was no evidence of an expansive process in the left ovary. The extension of examination to the upper abdomen revealed right renal agenesis and apparent compensatory hypertrophy of the left kidney (Figure 3).

### Patient 2

A 15-years-old woman presented with severe abdominal pain that occurred since the patient achieved menarche at 14 years of age; the patient also had irregular menstruation with severe dysmenorrhea and she reported that the pain worsened immediately prior to menstruation and improved with menstrual flow.

Upon physical examination, the findings were non-specific, with the exception of lower abdominal tenderness, arising from the pelvis, mildly tender to palpation. A gynecologic examination did not reveal any anomalies of her external genitalia or hymen.

So, MRI was then performed, using the same sequences protocol employed for Patient 1.

Contrast agent was not given and the patient's mother provided informed consent to perform MRI exam.

**Figure 4:**
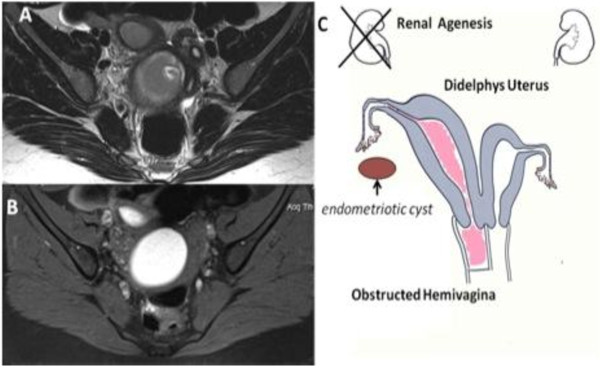
**Herlyn-Werner-Wunderlich syndrome (C) Drawing illustrates the triad**. A 15-years-old girl presenting the triad of didelphys uterus (class III MDA), an obstructed right hemivagina (class I MDA), and ipsilateral renal agenesis. (A) Axial turbo spin-echo T2 weighted MR image (B) Axial turbo spin-echo T1 fat-saturated weighted MR image showing centrally a wide hematocolpos (asterisk), corresponding to the obstructed right hemivagina. On the left side the uterus normally communicating with non-obstructed hemivagina.

**Figure 5:**
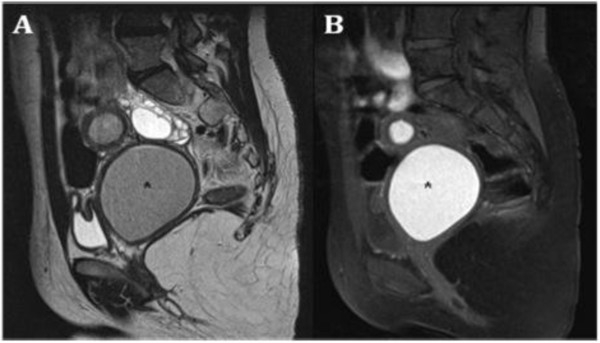
**A) Sagittal T2-weighted MR image demonstrates an enormously dilated hemivagina (asterisk) due to the presence of the transverse septum causing the obstruction and low T2-signal-intensity contents corresponding to a high T1-signal intensity; B) consistent with blood product due to the presence of haemoglobin**.

MRI indicated a uterine-vaginal malformation consisting of didelphys uterus communicating with a double vagina, of which the right vagina was obstructed (Figure 4). Consequently, there was a collection of fluid that exhibited a high signal intensity on T1 fat-saturated sequences, referred to as methaemoglobin, both in the right uterus (slightly enlarged: 18 mm diameter), and in the right obstructed vagina (which appears to be highly dilated and thus compressing the bladder, 60 × 65 × 76 mm) referred to hematocolpos (Figure 5).

**Figure 6:**
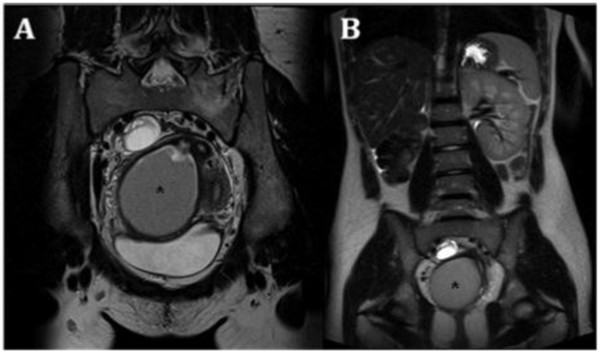
**A) Coronal T2 weighted images show the wide hematocolpos (asterisk)**. B) Trufi coronal image demonstrates the absence of right kidney, omolateral to the pelvic alteration, and a mild compensatory hypertrophy of the left kidney.

There was no evidence of an expansive process in the ovaries, but the extension of the examination to the upper abdomen showed right renal agenesis and apparent compensatory hypertrophy of the left kidney (Figure 6).

### Patient 3: an example of differential diagnosis

A 13-years-old female presented with severe abdominal pain. She reported that the pain had begun a few months earlier and had subsequently worsened over the past several weeks. The pain worsened immediately prior to menstruation and then improved once menstrual flow began. The patient also reported worsening constipation and denied problems with urination, nausea, vomiting or diarrhea.

The patient underwent menarche 2 months prior with menses exhibiting 3 to 4 days of very light flow. Physical examination did not reveal any alteration of her external genitalia. A previous sonography revealed a solitary left kidney as well as probable unicornuate uterus.

**Figure 7:**
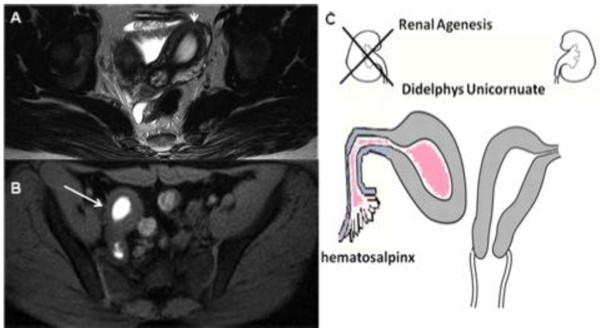
**A) TSE axial T2 weighted MR image**. B) Axial fat-saturated T1-weighted images. C) Drawing illustrates a 14-year-old girl with an unicornuate uterus and rudimentary right horn with an endometrial cavity that does not communicate with the uterus (class II MDA type) and shows an obstructed functional right uterine horn (long straight arrow) containing blood degradation products and a corresponding high T1fs-signal intensity, exhibiting no communication with the cavity of the normal left uterine horn (short straight arrow).

MRI examination confirmed an unicornuate uterus with a left single horn open into a normal vagina and a controlateral non-communicating rudimentary horn that contained material with a signal intensity similar to methaemoglobin (Figure 7).

**Figure 8:**
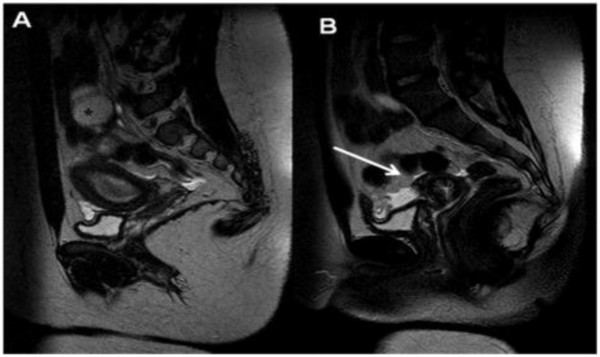
**A) Sagittal T2-weighted MR image shows a normal left uterine cavity communicating with a normal cervix (long straight arrow), which in turn communicates with a normal vagina**. (B) Right hematosalpinx (asterisk) with a distended non-communicating horn filled with fluid moderately hyperintense on T2 sequences due to haemoglobin degradation products.

**Figure 9:**
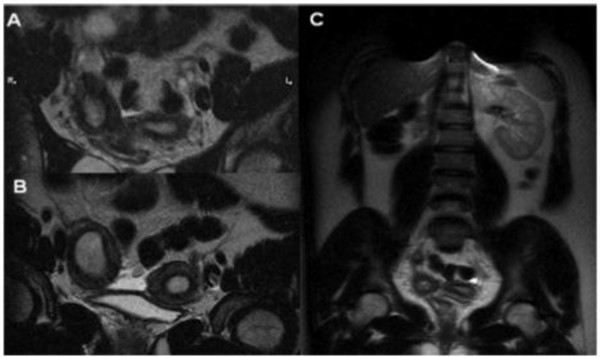
**A-B-C) Coronal T2 weighted images demonstrate the absence of right kidney, omolateral to the pelvic alteration**.

The ipsilateral Fallopian tube was characterized by fluid sero-mucinous distension (Figure 8). In the right paramedian side, at the level of the Douglas, posterior to the ideal point of continuity between uterus and rudimentary horn, there were some adjacent nodules most likely of endometrial etiology (14 mm, 38 mm in total) (Figure 9). In addition, a moderate rate of pelvic fluid was present.

The extension of the examination to the upper abdomen showed agenesis of the right kidney with compensatory hypertrophy of the left one and a left renal artery with a larger lumen, most likely of functional significance.

This is, evidently, another kind of MDA, different from the HWW syndrome, showing an association with uterine anomaly type IIb with renal aplasia, whereas HWW syndrome displays an association with Ia vaginal anomaly with a III uterine anomaly and renal aplasia.

## Conclusions

It could be desirable to achieve to an early diagnosis and treatment of HWW syndrome, in order to relieve acute symptoms, preserve normal fertility and prevent several medical complications. The correct diagnosis can be difficult due to the absence of specific findings upon physical examination and to non specific symptoms which delay the diagnosis.

Ultrasonography (US) is a very helpful tool in the diagnosis of Müllerian duct anomalies, infact the detection of haematocolpos, which appears as a fluid collection with low-level echoes, can make the diagnosis of genitor-urinary tract anomaly (didelphic uterus) easier, although it cannot identify the type of Müllerian ducts anomaly (MDA), whereas MRI, in cases when the differentiation of the uterine anomaly is not easily achievable by means of previous examination (physical examination, US, hysterosalpingography), is a suitable technique for the non-invasive evaluation of female pelvic anatomy because it provides more detailed information regarding uterine morphology, the continuity with each vaginal (obstructed and non-obstructed) lumen, and fluid content nature [12].

When a high suspicion of MDA exists, US should be performed initially to delineate any abnormalities in the genital tract, however, in order to obtain an accurate MDA classification, magnetic resonance imaging must be performed.

Although US, CT scan, and hysterosalpingography are commonly used for diagnosis because of their low cost, MRI is the most accurate method for diagnosis and it could help patients in maintaining fertility by allowing the most appropriate treatment option to be used [13].

In fact, in comparison to the other modalities, MRI is more sensitive in detecting the uterine contour, the shape of the intrauterine cavity, the character of the septum, as well as associated aspects such as endometriosis, pelvic inflammation and adhesions.

However, laparoscopy should now be considered the gold standard for the evaluation of female reproductive tract anomaly, although it could be reserved when the diagnosis is not clear after imaging or when MRI is not available and not performed as a routine procedure [7].

The decision to perform a laparoscopy is based on the following:

- the interval between menarche and diagnosis;

- the severity of the symptoms;

- the presence of a hematometra or pyometra.

Laparoscopy can be also therapeutic in some selected cases such as: drainage of hematocolpos/hematometrocolpos, septectomy, or marsupialization of the blind hemivagina. It is considered very helpful in order to reduce pelvic pain and lower the risk of infection and of further hematometra [1].

The outcome of pregnancy in these patients reveals that 87% go on to have a successful pregnancy, while abortions occur in 23% of the patients, 15% have preterm births, and 62% have full-term pregnancies and uncomplicated deliveries.

If surgery is not an immediate option for patients with HWW syndrome, menstrual suppression with combined oral contraceptive pills is advised to prevent further accumulation of hematocolpos and further hematometra [14,15].

The consequences that potentially occur due to the failure of treatment include urinary retention, hematosalpinx (blood in the fallopian tube), endometriosis and rupture of a tubo-ovarian abscess [16].

With each diagnosis of didelphys uterus in association with obstructed hemivagina on the lower abdomen MRI, HWW syndrome should be excluded and the exam should be extended to the upper abdomen to check for the presence of ipsilateral kidney.

Renal tract anomalies are associated with MDA in up to 30% of cases due to the close embryologic relationship between the paramesonephric and mesonephric ducts.

The most common renal tract anomaly associated with MDA is renal agenesis with right side prevalence [1,17,18]. Therefore, in all patients who present an MDA, it is imperative to investigate the urinary tract.

If renal agenesia is diagnosed, patients should have continuous follow-up to check for renal functioning as the risk of renal failure is very high [19]. Another very common condition in HWW syndrome patients is associated endometriosis.

The underlying pathophysiological mechanism is potentially the increased risk of retrograde menstruation: because of the obstructed vaginal outflow, the risk of endometrial tissue reflux through the fallopian tube, which can result in retrograde endometriosis, is higher, confirming the theory postulated by Sampson [20].

Several reports have confirmed that an association with endometriosis is present in obstructive Müllerian anomalies such as HWW syndrome, but it has not been confirmed to date in non-obstructive malformations. Therefore, it is imperative that MRI technology should be used to evaluate the presence of endometriosis.

Additionally, the presence of associated hematocolpos and hematometra, as well as the presence of an irregularly shaped or sized adnexal structure, or potentially expanding masses inside, need to be detected.

Another kind of obstructive Müllerian anomalies, different from the HWW syndrome, is OHVIRA syndrome, characterized by a variety of associated disorders. Infact, while the classic presentation includes obstructed hemivagina and ipsilateral renal anomaly (OHVIRA), some authors reported other renal anomalies, such as duplicated kidneys, dysplastic kidneys or retrovescical bands [21]. There have been also few reports of varied uterine anatomy; infact, a series of 42 cases of OHVIRA syndrome described, in 22% of cases, a septate uteri [15], but the majorority of cases are duplicated or didelphys uterus, although it has been described a case with two cervices, two uterine cavities and a single uterus [22].

## Consent

Written informed consent was obtained from the patient for publication of this case report and accompanying images. A copy of the written consent is available for review by the Editor-in-Chief of this journal.

## Abbreviations

HWW syndrome: Herlyn-Werner-Wunderlich syndrome; MDA: Müllerian Duct Anomalies; US: ultrasonography: MR: Magnetic Resonance; MRI: Magnetic Resonance Imaging; ASRM: American Society for Reproductive Medicine; CT: Computed Tomography.

## Competing interests

The authors declare that they have no competing interests.

## Authors' contributions

SB, VDP, RLC and CP analysed and interpreted the patient data. RDV, IS, RFG and BBZ performed the abdominal magnetic resonance. RDV, SB and VDP were the major contributors in writing the manuscript. All authors read and approved the final manuscript.

## Pre-publication history

The pre-publication history for this paper can be accessed here:

http://www.biomedcentral.com/1471-2342/12/4/prepub
